# Prevalence of unhealthy behaviors and their associations with non-suicidal self-injury, suicidal ideation and suicide attempt among Chinese adolescents

**DOI:** 10.1186/s13034-024-00742-y

**Published:** 2024-05-29

**Authors:** Wenjian Lai, Herui Wu, Liwen Yang, Ruiying Chen, Zhiyao Xin, Xiaojuan Zhang, Wanxin Wang, Lan Guo, Guoliang Huang, Ciyong Lu

**Affiliations:** 1 Department of Medical Statistics and Epidemiology, School of Public Health, Sun Yat-Sen university, 74 Zhongshan Rd 2, Guangzhou, 510080 China; 2 Center for Adverse Drug Reaction Monitoring of Guangdong, 753 Dongfeng East Road, Guangzhou, 510080 China

**Keywords:** Unhealthy behaviors, Non-suicidal self-injury, Suicidal ideation, Suicide attempt, Adolescent

## Abstract

**Background:**

Unhealthy lifestyle behaviors among adolescents have emerged as a significant public health concern worldwide, however, there is little investigation on the impact of unhealthy behaviors on non-suicidal self-injury (NSSI), suicidal ideation (SI) and suicide attempt (SA). This study aimed to investigate the prevalence of seven unhealthy behaviors as well as their associations with NSSI, SI and SA, and to explore whether the aforementioned associations differ across sex.

**Methods:**

A total of 74,152 adolescents were included in this study via a multi-stage, stratified cluster, random sampling method in 2021. Information about unhealthy behaviors (insufficient physical activity, current smoking, current drinking, excessive screen time, long homework time, insufficient sleep and unhealthy BMI), NSSI, SI, SA and other demographics was collected. Sampling weights were used to estimate the prevalence, and the weighted logistic regression models were performed. Stratified analyses by sex and sensitive analyses were conducted.

**Results:**

Overview, the weighted prevalence of adolescents had more than five unhealthy behaviors were 5.2%, with boys showing a higher prevalence than girls (6.5% vs.3.8%). Current smoking, current drinking, excessive screen use, long homework time, insufficient sleep, and unhealthy BMI were significantly associated with NSSI, SI and SA. Moreover, adolescents with high lifestyle risk scores were associated with an increased risk of NSSI (5–7 vs. 0: OR 6.38, 95% CI 5.24–7.77), SI (5–7 vs. 0: OR 7.67, 95% CI 6.35–9.25), and SA (5–7 vs. 0: OR 9.57, 95% CI 6.95–13.17). Significant sex differences were found in the associations of unhealthy behaviors with NSSI, SI and SA.

**Conclusion:**

Unhealthy behaviors are quite common among Chinese adolescents. Adolescents with multiple unhealthy behaviors are associated with increased risks of NSSI, SI, and SA. The implementation of school and family-based interventions to promote healthy lifestyles is recommended as a preventive measure against self-injurious behavior and suicidality in adolescents.

**Supplementary Information:**

The online version contains supplementary material available at 10.1186/s13034-024-00742-y.

## Introduction

Suicide is the fourth leading cause of death among adolescents according to World Health Organization (WHO) [1], placing a tremendous burden on societies and families. Non-suicidal self-injury (NSSI), suicidal ideation (SI) and suicide attempt (SA) are important predictors of future suicide and pose serious public health concerns for adolescents worldwide [2–5]. Previous evidence indicated that 26.1%, 17.5%, 4.4% of Chinese adolescents experienced NSSI, SI, and SA within the past 12 months, respectively [6], highlighting the urgent need to address self-injurious behavior and suicidality in adolescents. Therefore, it is vital to identify modifiable factors associated with NSSI, SI and SA to effectively prevent suicide in adolescents.

Recent studies have emphasized the links between unhealthy lifestyle behaviors and self-injurious behavior and suicidality [4, 7, 8]. Adopting a healthy lifestyle, which includes regular physical activity, avoiding smoking, limiting alcohol consumption, and ensuring sufficient sleep, plays a crucial role in promoting both physical and mental well-being [9]. Adolescence is a critical period of transitioning from childhood to adulthood, during which lifestyle habits are formed and developed [10]. Consequently, unhealthy lifestyle behaviors among adolescents have emerged as an important public health concern. Inadequate sleep, lack of physical activity, and excessive screen time are common lifestyle risk behaviors in adolescents recently [9, 11, 12]. It is recommended that adolescents aim for 8–10 h of sleep per night [13], however, a majority of Chinese adolescents sleep less than eight hours [14]. Although WHO recommends an average of 60 min/day of moderate-to-vigorous intensity physical activity throughout the week for adolescents [15], a cross-sectional study conducted in China revealed that 65.9% of adolescents are physically inactive [16]. With the advancement of technology, screen usage, including watching TV, tablets, and smartphones, has also become an integral part of daily life. It is advised that adolescents limit their screen time to less than 2 h per day [17], however, most adolescents exceed this recommendation [18]. Additionally, smoking and drinking have been reported among Chinese adolescents [4, 19].

There is growing attention on the associations of unhealthy behaviors with NSSI, SI and SA among adolescents. Previous studies have demonstrated that unhealthy BMI status, smoking and drinking, may increase the vulnerability of adolescents to engage in suicidal behaviors [19, 20]. Moreover, excessive time spent on sedentary behaviors, such as screen time and homework time, has been associated with suicidal behavior in adolescents [21, 22]. Additionally, inadequate sleep and lack of physical activity have been widely reported to be associated with suicidal behavior in adolescents [14, 23].

However, most previous studies only consider the independent association of each unhealthy behavior with NSSI, SI and SA [7, 14, 19, 22], while ignoring the impact of co-occurrence of multiple unhealthy behaviors. Furthermore, previous evidence has suggested that there are sex differences in the distribution of unhealthy behaviors among adolescents, with boys spending more time on physical activity and screens compared to girls [16, 24, 25]. Additionally, adolescent boys and girls exhibit different characteristics in terms of suicidal behavior [4, 8, 20], therefore, there is a great need to explore the associations of unhealthy behaviors with NSSI, SI and SA in different sex, which is critical for sex-specific risk identification and intervention.

Herein, to gain a better understanding of the impact of unhealthy behaviors on suicidal behaviors, we conducted this study aimed to investigate the prevalence of seven unhealthy behaviors as well as their associations with NSSI, SI and SA, and to explore whether the aforementioned associations differ between adolescent boys and girls.

## Method

### Study design and participants

The study utilized the data from the 2021 School-based Chinese Adolescents Health Survey (SCAHS), which is a large-scale survey about the health-related behaviors and mental health problems among Chinses adolescents (grades 7–12) conducted from October 2021 to March 2022. A multi-stage, stratified cluster, random sampling method was used in the 2021 SCAHS to obtain a nationally representative sample of Chinese adolescents, and the procedures have been descried in previous studies [19, 26]. In stage 1, the provinces in mainland China were divided into three economic strata (high, medium, low) based on per capita gross domestic product (GDP) level, and three provinces were randomly selected from each stratum (only two provinces from medium level were selected; see Fig. S1 for the geographical distribution of the selected provinces). A total of eight provinces were selected. In stage 2, we divided the cities within each selected province into three economic strata (high, medium, low) according to per capita GDP level, and one city from each stratum was randomly chosen. In stage 3, six junior high schools, four senior high schools, and two vocational high schools were randomly chosen based on the proportion of the three types of schools in the selected cities. In stage 4, two classes were randomly selected from each grade within the chosen schools, and all students in the selected classes were invited to participated in the study voluntarily.

To protect the privacy of the students, information was collected via completing anonymous self-reported questionnaires by students in the classroom during a regular class period (i.e., 45 min), and the survey was administrated by the trained research assistants without the presence of teachers. Prior to the survey, the assistants informed all participants about the purpose of the study in detail, and the completeness and quality of the questionnaires were also checked by the assistants at the end of survey.

In total, 85,046 students aged 11–21 years were invited on the day of the survey, and 74,152 students’ questionnaires were completed and qualified for our study (a response rate of 87.2%).

The study was conducted in accordance with the Declaration of Helsinki, and was approved by Sun Yat-sen University School of Public Health Institutional Review Board. Written informed consents were obtained from each participant who was at least 18 years old or from one of the parents of each participant who was under 18 years old.

### Measure

#### Non-suicidal self-injury (NSSI)

NSSI was measured by the Chinese version of the Functional Assessment of Self-Mutilation, which has been widely used in Chinese adolescents [6, 27]. The scale consisted of eight different forms of NSSI (i.e., hitting, head banging, stabbing, pinching, scratching, biting, burning, and cutting), and respondents were asked to report the frequency they harmed themselves deliberately in the above forms without the thought of taking the life during the past 12 months. Students who reported the frequency of NSSI of three or more times were classified as having NSSI [6].

#### Suicidal ideation and suicidal attempt

SI was assessed by the question “During the past 12 months, how many times did you seriously consider attempting suicide?” SA was measured by the question “During the past 12 months, how many times did you actually attempt suicide?” For the above questions, students who reported one or more times were identified as having SI or SA.

### Unhealthy behaviors

Unhealthy behaviors included insufficient physical activity, current smoking, current drinking, excessive screen time, long homework time, insufficient sleep and unhealthy body mass index (BMI).

Physical activity was measured by the question “During the past week, how many days did you exercise that increased your heart rate or breathing rate for at least 60 min?” The response ranged from 0 to 7, and adolescents who reported less than 5 days were identified as having insufficient physical activity [16].

Current smoking/drinking was assessed by asking the adolescents how many days they smoke/drank alcohol during the last 30 days, and those answered one or more days were classified as current smoking/drinking.

Time spent on screen was investigated by self-reported time spent on a typical weekday and weekend day performing a variety of screen-based activities for entertainment, including surfing the Internet (e.g., video watching), watching TV and playing electronic devices (e.g., smartphone, tablet). Average daily time was calculated using the following formula: (weekday time*5 + weekend time*2)/7 [18]. Adolescents who spent screen time more than 2 h per day were identified as having excessive screen time [18, 28].

Time spent on homework was also investigated by self-reported time spent on a typical weekday and weekend day doing homework, and the average daily time was calculated based on the above formula. Adolescents who spent homework time more than 3 h per day were classified as having long homework time [9].

Sleep duration was measured by asking adolescents how many hours they slept on weekdays and weekends during the last month, respectively. Daily sleep duration was calculated by the formula: (hours on weekdays*5 + hours on weekends*2)/7. Adolescents who reported sleep duration less than 8 h per day were identified as having insufficient sleep [14].

BMI was measured by self-report height and weight. Based on body mass index growth curves for Chinese children and adolescents [29], BMI was classified into four categories: normal, underweight (BMI < 5th), overweight (85th ≤ BMI < 95th), obesity (BMI ≥ 95th). BMI was also binned into a dichotomous variable indicating healthy (normal) or unhealthy (underweight, overweight and obesity) [30].

To evaluate the impact of multiple unhealthy behaviors on mental health, a lifestyle risk score was constructed. For the above seven behaviors, respondents were scored 1 if engaged in each of the unhealthy behaviors and 0 if they did not. Therefore, the lifestyle risk score was a sum of each score, with the score ranging from 0 to 7.

### Other variables

Other variables included age, biological sex (male, female), residence (urban, rural), having brothers or sisters (yes, no), living arrangement (living with parents, living with a single parent, living with others), household socioeconomic status (HSS; good, average, poor), and parents’ education level (primary school or below, middle school, university or above).

### Statistical analysis

Considering the intricate multistage sampling design, for all statistical analyses, data were weighted via appropriate sampling weights to adjust for unequal probabilities of selection. In the current study, the missing data of the potential covariates were imputed using multiple imputations by chained equations.

First, descriptive analyses stratified by NSSI, SI and SA, respectively, were conducted to describe the demographic characteristics. Data were presented as mean (standard deviation, SD) for continuous variables and frequencies (proportions) for categorical variables, and the differences among groups were tested using appropriate statistical approaches such as *t*-test or Chi-square test. Second, the weighted prevalence of each lifestyle risk behavior among Chinese adolescents was estimated, and differences in distribution between boys and girls were also compared. Third, weighted logistic regression models were used to estimate the odds ratio (OR) and 95% confidence interval (CI) to explore the association of lifestyle risk behavior with NSSI, SI and SA. Two models were conducted to assess the impact of potential covariates based on previous literature. Model 1 was adjusted for age and sex. Model 2 was adjusted for age, sex, province, residence, having brothers/sisters, living arrangement, HSS, father’s education level, and mother’s education level.

Subgroup analyses were also conducted to examine whether the associations of lifestyle risk behavior with NSSI, SI and SA varied by sex, and any sex difference in the associations were also examined via calculating a ratio of ORs. In sensitivity analyses, we repeated all analyses using the complete dataset to test the robustness of the results.

All data analyses were carried out using R 4.1.0 (the R Foundation for Statistical Computing, Vienna, Austria), and the statistical tests were two-sided, with *P* < 0.05 considered statistically significant.

## Result

### Sample characteristic

Table 1 presents the sample characteristics stratified by NSSI, SI and SA. The study included 74,152 adolescents in the final analyses, with a mean (SD) age of 14.7 (1.8) years, and almost half were boys (weighted prevalence, 52.6%). The weighted prevalence of NSSI, SI and SA was 14.6%, 17.6% and 5.2%, respectively. Adolescents with NSSI, SI and SA were more likely to be girls, from urban residences, living with a single parent or others, have poor HSS than those without NSSI, SI and SA.

**Table 1:** Sample characteristic

Variable	Overall	NSSI			Suicidal ideation			Suicidal attempt		
		Yes	No	P value	Yes	No	P value	Yes	No	P value
Total	74,152 (100)	11,115 (100)	63,037 (100)		13,286 (100)	60,866 (100)		4047 (100)	70,105 (100)	
Age, mean (SD)	14.7 (1.8)	14.6 (1.7)	14.7 (1.8)	< 0.001	14.5 (1.7)	14.7 (1.8)	< 0.001	14.3 (1.6)	14.7 (1.8)	< 0.001
Sex										
Male	36,895 (52.6)	4084 (39.1)	32,811 (54.9)	< 0.001	4655 (37.2)	32,240 (55.9)	< 0.001	1207 (32.3)	35,688 (53.8)	< 0.001
Female	37,257 (47.4)	7031 (60.9)	30,226 (45.1)		8631 (62.8)	28,626 (44.1)		2840 (67.7)	34,417 (46.2)	
Province										
Guangdong	10,159 (22.0)	1312 (19.3)	8847 (22.5)	< 0.001	1771 (21.9)	8388 (22.0)	< 0.001	398 (16.8)	9761 (22.3)	< 0.001
Chongqing	9448 (6.1)	1569 (7.0)	7879 (6.0)		1980 (7.3)	7468 (5.9)		580 (7.4)	8868 (6.1)	
Shandong	8901 (18.1)	1164 (16.4)	7737 (18.4)		1289 (15.1)	7612 (18.8)		412 (16.3)	8489 (18.2)	
Liaoning	8977 (6.0)	1174 (5.5)	7803 (6.1)		1402 (5.4)	7575 (6.1)		426 (5.6)	8551 (6.0)	
Henan	9733 (23.6)	1493 (24.7)	8240 (23.4)		1791 (24.8)	7942 (23.4)		520 (24.8)	9213 (23.5)	
Yunnan	10,875 (10.1)	2011 (12.2)	8864 (9.7)		2076 (10.4)	8799 (10.0)		675 (11.4)	10,200 (10.0)	
Guizhou	8294 (9.6)	1379 (11.1)	6915 (9.4)		1678 (11.2)	6616 (9.3)		594 (13.0)	7700 (9.4)	
Heilongjiang	7765 (4.5)	1013 (3.8)	6752 (4.6)		1299 (4.0)	6466 (4.6)		442 (4.8)	7323 (4.4)	
Residence										
Urban	34,971 (48.2)	5478 (50.7)	29,493 (47.8)	< 0.001	6684 (52.5)	28,287 (47.3)	< 0.001	2069 (53.4)	32,902 (47.9)	< 0.001
Rural	39,181 (51.8)	5637 (49.3)	33,544 (52.2)		6602 (47.5)	32,579 (52.7)		1978 (46.6)	37,203 (52.1)	
Having brothers/sisters										
Yes	53,955 (78.0)	8245 (79.0)	45,710 (77.9)	0.013	9857 (78.8)	44,098 (77.9)	0.032	3022 (79.3)	50,933 (78.0)	0.068
No	20,197 (22.0)	2870 (21.0)	17,327 (22.1)		3429 (21.2)	16,768 (22.1)		1025 (20.7)	19,172 (22.0)	
Living arrangement										
Living with parents	48,620 (66.8)	6557 (60.4)	42,063 (67.9)	< 0.001	7690 (59.4)	40,930 (68.4)	< 0.001	2246 (57.3)	46,374 (67.3)	< 0.001
Living with a single parent	13,903 (18.3)	2515 (22.0)	11,388 (17.7)		3137 (23.0)	10,766 (17.3)		996 (23.9)	12,907 (18.0)	
Living with others	11,629 (14.8)	2043 (17.6)	9586 (14.4)		2459 (17.6)	9170 (14.3)		805 (18.8)	10,824 (14.6)	
HSS										
Good	18,931 (27.8)	2394 (23.3)	16,537 (28.6)	< 0.001	2806 (23.1)	16,125 (28.8)	< 0.001	946 (25.1)	17,985 (27.9)	< 0.001
Average	44,252 (59.4)	6571 (59.7)	37,681 (59.3)		7867 (59.6)	36,385 (59.3)		2258 (56.6)	41,994 (59.5)	
Poor	10,969 (12.9)	2150 (17.0)	8819 (12.1)		2613 (17.3)	8356 (11.9)		843 (18.2)	10,126 (12.6)	
Father’s education level										
Primary school or below	44,050 (56.4)	6636 (56.8)	37,414 (56.3)	< 0.001	7906 (56.3)	36,144 (56.4)	0.106	2419 (56.3)	41,631 (56.4)	0.896
Middle school	17,071 (24.3)	2397 (22.7)	14,674 (24.6)		2998 (23.7)	14,073 (24.5)		924 (24.7)	16,147 (24.3)	
University or above	13,031 (19.3)	2082 (20.6)	10,949 (19.1)		2382 (20.0)	10,649 (19.1)		704 (19.1)	12,327 (19.3)	
Mother’s education level										
Primary school or below	46,811 (60.5)	6985 (60.0)	39,826 (60.6)	0.059	8373 (59.8)	38,438 (60.7)	0.008	2569 (60.5)	44,242 (60.5)	0.930
Middle school	15,556 (22.0)	2276 (21.6)	13,280 (22.1)		2743 (21.6)	12,813 (22.1)		838 (21.8)	14,718 (22.0)	
University or above	11,785 (17.5)	1854 (18.4)	9931 (17.3)		2170 (18.6)	9615 (17.2)		640 (17.7)	11,145 (17.5)	

All numbers were unweighted, whereas all percentages were adjusted for sampling weights

*HSS* household socioeconomic status, *NSSI* non-suicidal Self-Injury

### Prevalence of unhealthy behaviors among Chinese adolescents

**Fig. 1:**
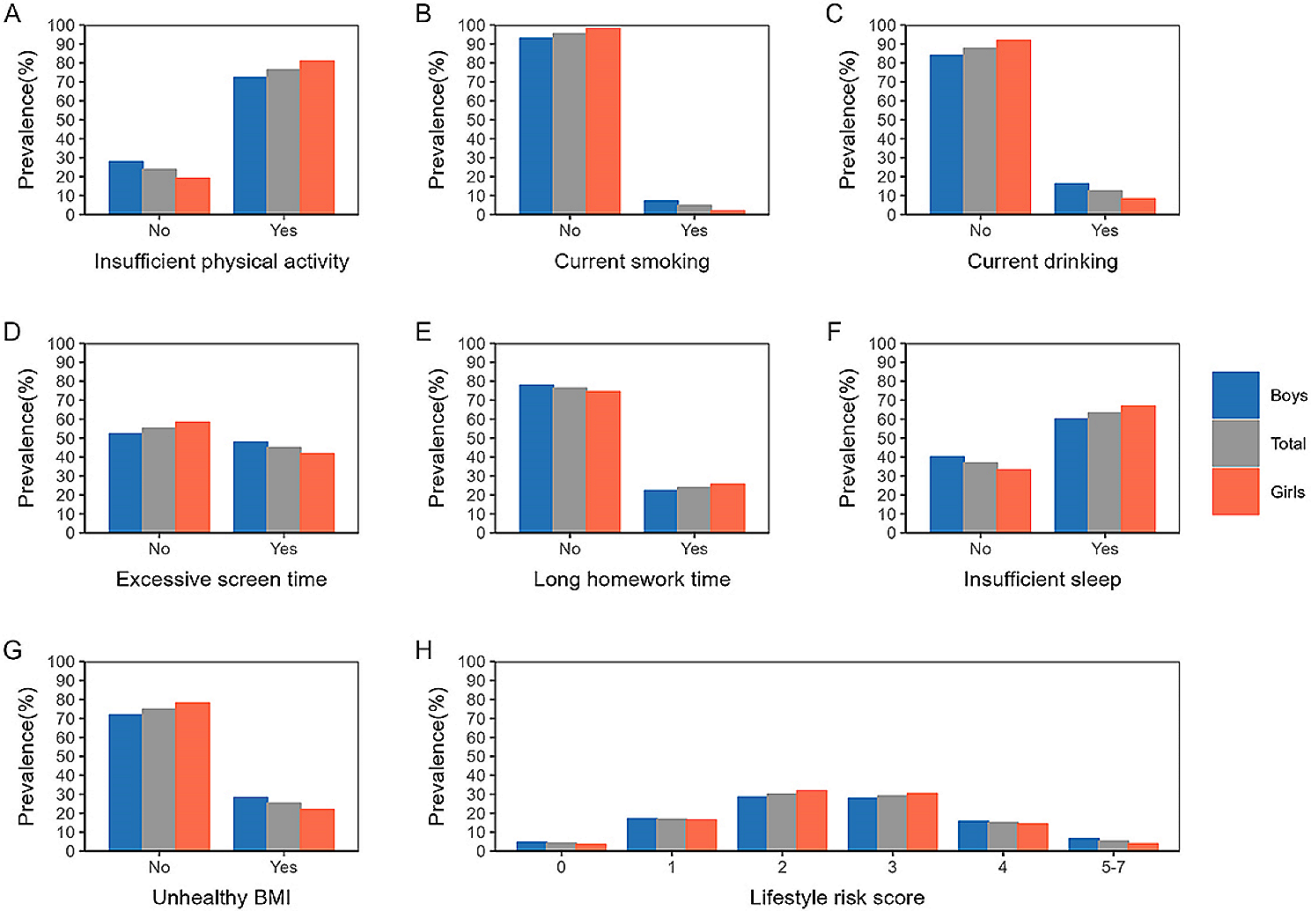
The weighted prevalence of unhealthy behaviors among Chinese adolescents

Sex-specific distribution of unhealthy behaviors are presented in Fig. 1 and Table S1. The weighted prevalence of adolescents had zero or more than five unhealthy behaviors was 4.1 and 5.2%, respectively. The most common unhealthy behaviors among Chinese adolescents were insufficient physical activity (76.3%), followed by insufficient sleep (63.2%), excessive screen time (44.9%), unhealthy BMI (25.2%), long homework time (23.8%), current drinking (12.4%) and current smoking (4.7%). There were significant differences in the distribution of unhealthy behaviors between boys and girls, where boys were more likely to have excessive screen time, unhealthy BMI, current drinking and current smoking.

### Association of unhealthy behaviors with NSSI, SI and SA

As presented in Table 2, the weighted prevalence of NSSI, SI, and SA among Chinese adolescents with more than five unhealthy behaviors was 24.7%, 30.7% and 11.5%, respectively. After adjusting for covariates in Model 2, adolescents with high lifestyle risk scores were associated with an increased risk of NSSI (5–7 vs. 0: OR 6.38, 95% CI 5.24–7.77), SI (5–7 vs. 0: OR 7.67, 95% CI 6.35–9.25), and SA (5–7 vs. 0: OR 9.57, 95% CI 6.95–13.17). A trend on the association of the number of unhealthy behaviors with NSSI, SI and SA was also observed (*P* for trend < 0.001).

**Table 2:** The weighted associations of lifestyle risk score with NSSI, suicidal ideation and suicidal attempt among Chinese adolescents

Outcome	Lifestyle risk score	Weighted prevalence of outcomes, %	Model 1		Model 2		*P* for trend*
			OR (95% CI)	*P*	OR (95% CI)	*P*	
NSSI							< 0.001
	0	6.1 (5.1-7.0)	1.00 (Reference)		1.00 (Reference)		
	1	8.8 (8.2–9.4)	1.50 (1.24, 1.82)	< 0.001	1.47 (1.21, 1.78)	< 0.001	
	2	12.3 (11.8–12.8)	2.30 (1.92, 2.76)	< 0.001	2.21 (1.84, 2.65)	< 0.001	
	3	16.7 (16.1–17.3)	3.52 (2.93, 4.22)	< 0.001	3.37 (2.81, 4.05)	< 0.001	
	4	20.4 (19.5–21.3)	4.81 (3.99, 5.79)	< 0.001	4.57 (3.80, 5.51)	< 0.001	
	5–7	24.7 (23.2–26.2)	6.88 (5.65, 8.38)	< 0.001	6.38 (5.24, 7.77)	< 0.001	
Suicide ideation							< 0.001
	0	7.2 (6.1–8.3)	1.00 (Reference)		1.00 (Reference)		
	1	10.1 (9.5–10.8)	1.45 (1.21, 1.74)	< 0.001	1.43 (1.19, 1.71)	< 0.001	
	2	15.0 (14.4–15.5)	2.42 (2.03, 2.88)	< 0.001	2.33 (1.95, 2.77)	< 0.001	
	3	19.7 (19.1–20.4)	3.67 (3.09, 4.37)	< 0.001	3.49 (2.93, 4.15)	< 0.001	
	4	25.1 (24.2–26.1)	5.45 (4.56, 6.50)	< 0.001	5.14 (4.30, 6.15)	< 0.001	
	5–7	30.7 (29-32.3)	8.24 (6.83, 9.95)	< 0.001	7.67 (6.35, 9.25)	< 0.001	
Suicide attempt							< 0.001
	0	2.0 (1.4–2.5)	1.00 (Reference)		1.00 (Reference)		
	1	2.7 (2.3-3.0)	1.38 (1.00, 1.90)	0.051	1.34 (0.97, 1.84)	0.076	
	2	4.2 (3.9–4.5)	2.43 (1.79, 3.29)	< 0.001	2.32 (1.71, 3.15)	< 0.001	
	3	5.7 (5.4–6.1)	3.74 (2.76, 5.08)	< 0.001	3.57 (2.63, 4.84)	< 0.001	
	4	7.8 (7.3–8.4)	5.81 (4.26, 7.91)	< 0.001	5.46 (4.00, 7.44)	< 0.001	
	5–7	11.5 (10.4–12.6)	10.50 (7.63, 14.45)	< 0.001	9.57 (6.95, 13.17)	< 0.001	

Model 1 adjusted for age, sex

Model 2 adjusted for age, sex, province, residence, having brothers/sisters, living arrangement, HSS, and parents’ education level

*NSSI* Non-suicidal Self-Injury

*P for trend was estimated based on Model 2

**Fig. 2:**
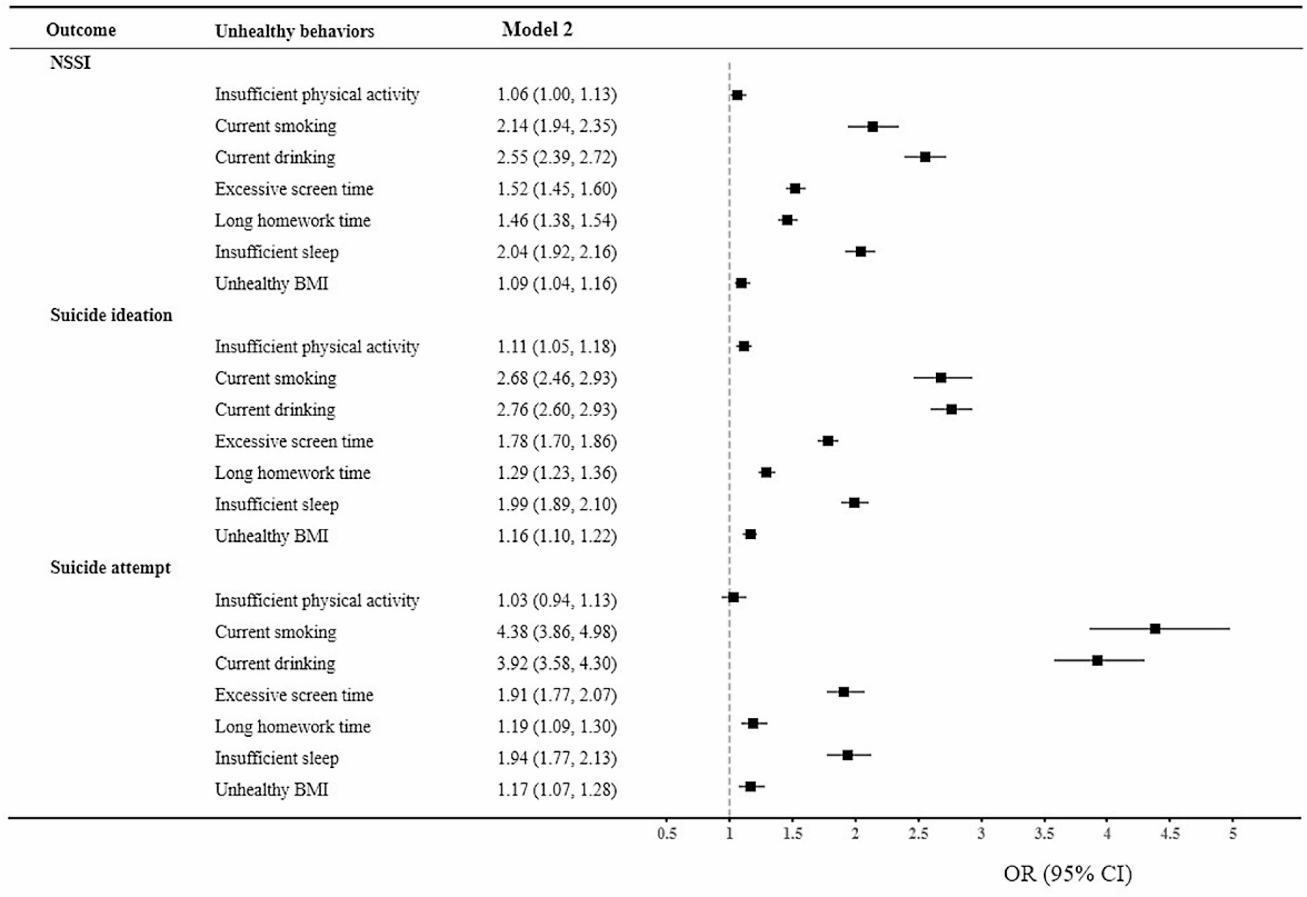
The weighted associations of specific unhealthy behavior with NSSI, suicidal ideation and suicidal attempt among Chinese adolescents. Model 2 adjusted for age, sex, province, residence, having brothers/sisters, living arrangement, HSS, and parents’ education level

For each unhealthy behavior, after adjusting for covariates in Model 2, current smoking, current drinking, excessive screen time, long homework time, insufficient sleep, and unhealthy BMI were significantly associated with NSSI, SI and SA. In addition, insufficient physical activity was only associated with NSSI and SI (shown in Fig. 2 and Table S2).

### Subgroup analyses

**Fig. 3:**
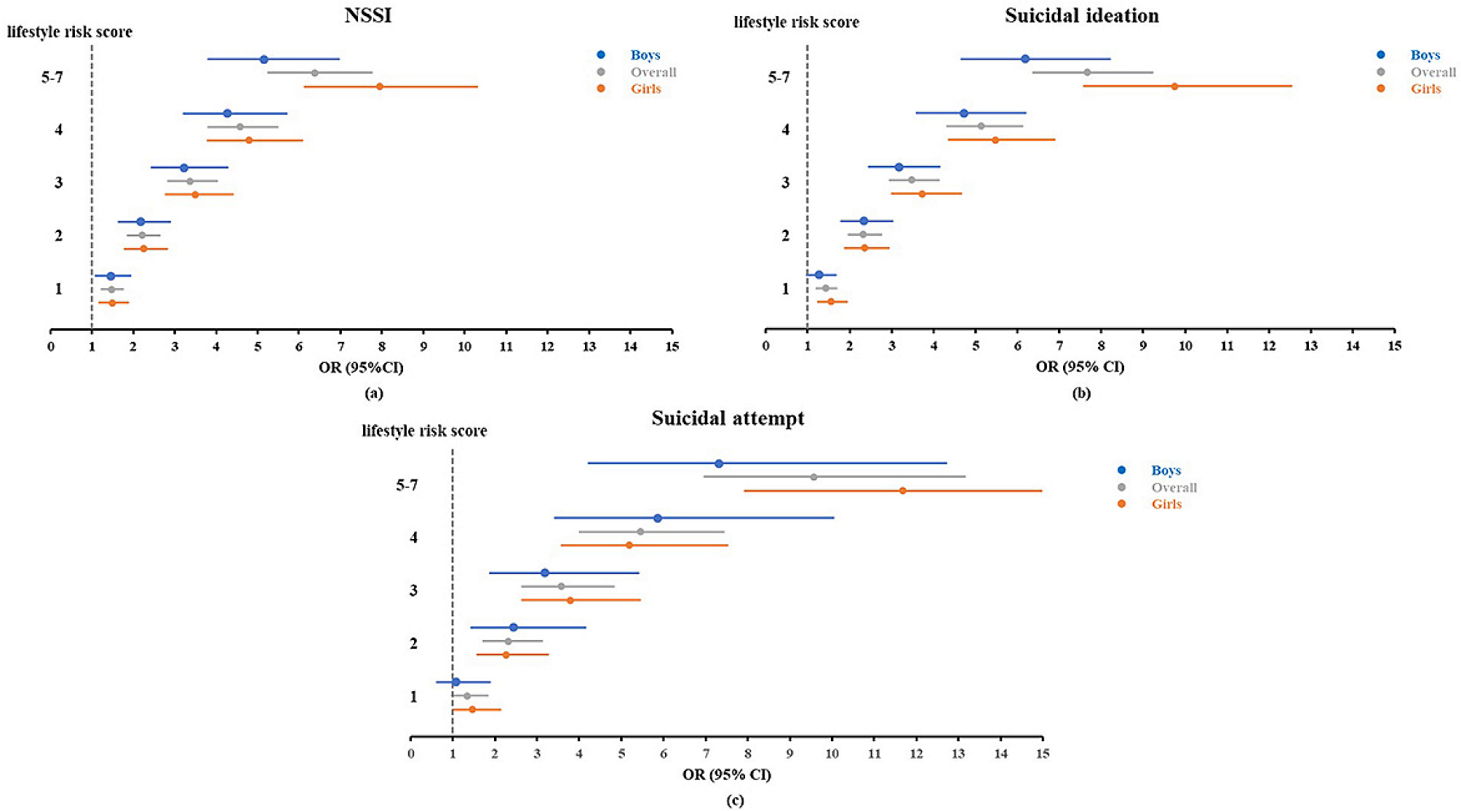
The weighted associations of lifestyle risk score with NSSI, suicidal ideation and suicidal attempt among boys and girls. All models were adjusted for age, sex, province, residence, having brothers/sisters, living arrangement, HSS, and parents’ education level (models for boys and girls were not adjusted for sex)

The results stratified by sex were shown in Fig. 3. After adjusting for covariates, the significant associations of lifestyle risk scores with NSSI, SI and SA were also found among boys and girls. Adolescent girls who had more than 5 unhealthy behaviors was associated with higher odds of NSSI (OR 7.95, 95% CI 6.12–10.32) and SI (OR 9.75, 95% CI 7.57–12.55) than boys, and significant sex difference were found for the above associations (*P* for ROR < 0.05; Table S3).

Regarding each unhealthy behavior, girls who engaged in current smoking, current drinking was associated with a higher risk of NSSI, SI and SA compared to boys. In addition, the association between long homework time and NSSI was stronger among boys, while the association between excessive screen time and SI was stronger in girls (Table S4).

### Sensitivity analyses

Sensitivity analyses using complete dataset (*n* = 64,716) to repeat the main analyses to examine the robustness of the results. The main analyses with complete dataset showed that the associations of lifestyle risk score with NSSI, SI and SA remained essentially unchanged (Table S5).

## Discussion

A large-scale, school-based survey was conducted to investigate the prevalence of seven unhealthy behaviors and their associations with NSSI, SI and SA. The data revealed that 5.2% of Chinese adolescents had more than five unhealthy behaviors, and the prevalence among boys (6.5%) was higher than girls (3.8%). Unhealthy behaviors, including current smoking, current drinking, excessive screen time, long homework time, insufficient sleep and unhealthy BMI status, had a negative impact on self-injurious behavior and suicidality. Moreover, adolescents with high lifestyle risk scores were associated with an increased risk of NSSI, SI, and SA, and girls showed stronger association than boys.

Although only 5.2% of adolescents had more than five unhealthy behaviors, the majority of them experienced physical inactivity (76.3%), sleep deficiency (63.2%), and excessive screen time (44.9%). Similarly, the US Youth Risk Behavior Survey (YRBS) had shown that 77.9% of US adolescents slept less than 8 h per day and 76.8% of them did not get sufficient physical activity [7]. Another cross-sectional study conducted in Australia had found that most adolescents exceeded guidelines for screen time (84%), did not achieve sufficient moderate-to-vigorous physical activity (75%) and got inadequate sleep (56%) [31]. Discrepancies in the prevalence of these behaviors may be attributed to cultural backgrounds or different characteristics of the study populations, such as different sampling methods, measurements, and criteria. Consistent with previous evidence [12, 14, 19], the current study also found that adolescent boys and girls show different patterns in the distribution of unhealthy behaviors. Boys were more likely to smoke, drink, spend excessive time on screen-based activities, and have an unhealthy BMI status, while girls were more likely to be physically inactive, spend too much time on homework, and not get enough sleep. Despite the low rate of having more than five unhealthy behaviors, the negative impact of these behaviors emphasizes the need for schools and families to assist adolescents in developing healthy lifestyle habits to improve their well-being.

In line with previous evidence, this study suggested that excessive screen time was associated with NSSI and suicidality among adolescents. Jin and colleagues conducted a cross-sectional study involving 16,853 Chinese adolescents and found that those who spent more than 2 h per day on screen had an increased risk of SI and SA [8]. Similarly, a separate cross-sectional study conducted in Canada indicated that screen time ≤ 2 h/d had a protective effect against suicidality among adolescents [28]. Excessive screen time may lead adolescents to become immersed in a virtual world where rules may not apply, potentially increasing the likelihood of behavioral problems such as aggressive behaviors [32]. Notably, the study found that the association between screen time and SI was stronger in girls. This could be attributed to the fact that females tend to adopt avoidant and inactive coping styles, and previous research has reported a higher prevalence of emotional problems among female adolescents [33]. The accumulation of negative emotions after prolonged screen time may therefore make them more susceptible to engaging in suicidal behavior [27].

Our findings indicated that insufficient sleep duration was associated with NSSI, SI and SA, which was consistent with previous literature. Similarly, YRBS has demonstrated that US adolescents who sleep less than 8 h per day have a higher risk of SI and SA [34]. The possible explanation may be that sleep deprivation exerts a negative impact on adolescents’ attention, judgment and impulse control, which contributes to self-injurious behavior and suicidality due to impulsivity and loss of inhibition [35, 36]. Additionally, our study found an association between unhealthy BMI status and suicidal behaviors, which aligns with previous evidence. A meta-analysis including 104,907 adolescents in 45 low- and middle-income countries also suggested that overweight and obesity were significantly associated with SI and SA [37]. Kim et al. indicated that adolescents being underweight were significantly associated with increased odds of having a suicidal attempt [38]. Given the current popularity of fitness among adolescents, adolescents being obese may be perceived as lacking self-control, while those being underweight may be stigmatized as skinny [39, 40]. The negative attitudes towards obesity and the stigma faced by those identified as underweight can influence self-esteem during adolescence, resulting in poor mental health [19, 41]. Consequently, adolescents with an unhealthy BMI status may experience more social support frustration and self-stigma, leading to an elevated risk of suicidal behavior. However, we did not observe a significant sex difference in the associations of sleep and BMI with NSSI, SI and SA.

In addition, the study found that both current smoking and current drinking were positively associated with suicidal behaviors among adolescents. Several studies found similar associations corroborating [42, 43]. It is well established that smoking and excessive alcohol consumption can disturb the neuroendocrine system, which subsequently increases the risk of suicide-related behaviors [44, 45]. Previous evidence has also suggested that smoking, drinking, and self-injurious behavior share similar psychological processes, and substance use may contribute to the habituation of self-injurious behavior [46]. Interestingly, this study found that the associations of current smoking and drinking with NSSI, SI and SA were stronger in adolescent girls compared to boys, which supports prior evidence. Similarly, a cross-sectional study conducted in Korean adolescents also showed that current smoking had a greater impact on suicidal behaviors among female adolescents than males [43]. Likewise, Phillips et al. also found that the associations between alcohol use and suicidality were stronger in adolescent girls than boys [47].

Despite the extensive body of research on the associations of unhealthy behaviors with self-injurious behavior and suicidality, there are few studies focusing on the number of unhealthy behaviors on suicidal behaviors while considering a range of covariates. It is worth noting that the occurrence of multiple unhealthy behaviors has a more detrimental impact on suicidal behaviors compared to single behavior, as supported by previous studies. Engaging in a single unhealthy behavior may increase the likelihood of engaging in other unhealthy behaviors, and when these risky behaviors are combined, the possibility of mental health problems increases [48]. For instance, Hwang et al. suggested that adolescents with a higher lifestyle risk score were more prone to suicidal ideation [30], and a similar finding was also observed in Australian adolescents [49]. The probability of mental health problems increases with the number of unhealthy behaviors, providing strong evidence for population-level interventions for integrated unhealthy lifestyle behaviors. Additionally, we could observe significant sex differences in the foregoing associations, which was consistent with prior research [9]. This study revealed that a considerable number of adolescents engaged in multiple unhealthy behaviors simultaneously, and the results also suggested that the co-occurrence of multiple risk factors heightens the likelihood of adverse health outcomes [50, 51]. In sum, this study highlights the importance of not only considering the impact of specific unhealthy behavior on self-injurious behavior and suicidality among adolescents, but also recognizing the co-occurrence of multiple risk behaviors. The findings of this study contribute to the understanding of the necessity for school and family-based health promotion efforts and the development of strategies to prevent self-injurious and suicidal behaviors in adolescents by promoting healthy lifestyles, ultimately improving their quality of life.

The main strength of our study is that our study uses a multi-stage, stratified cluster, random sampling method to obtain a nationally representative sample of Chinese adolescents, which provides sufficient statistical power and broad representativeness to investigate the prevalence of multiple unhealthy behaviors and their associations with NSSI, SI and SA. However, several limitations are worth noting. First, the information about unhealthy behaviors, self-injurious behavior and suicidality was collected via self-report, which can not preclude recall bias. Meanwhile, despite the survey was anonymous, adolescents may provide answers influenced by social desirability because of the sensitivity to such questions (such as current smoking and suicidal behaviors). However, previous evidence has demonstrated that self-reporting is a common and widely accepted method [52]. Second, this study did not include diet-related behaviors (e.g., breakfast and beverage consumption), which is also an important part of lifestyle behavior. Future studies need to consider the impact of these behaviors. Third, the study sample was primarily derived from school students and did not include adolescents absent from school, nevertheless, suicidal behaviors may be more common among those absent students. Fourth, the causality of the assessed factors is difficult to determine since the nature of current research is a cross-sectional study design. Given that the measurement of outcome (NSSI, SI, SA) was limited to last 12 months while the measurement of partial exposure (e.g. current smoking/drinking) was limited to the past month, the possibility of potential reverse causation also exists. Fifth, the study was conducted during the COVID-19 pandemic, so the results might be biased due to the influence of COVID-19. Therefore, there is a need for future studies to remove the effect of COVID-19 and verify our findings.

## Conclusion

The results of this study demonstrate that multiple unhealthy behaviors are quite common among adolescents. Furthermore, this study expands upon existing evidence by highlighting the association of high lifestyle risk scores with NSSI, SI, and SA in adolescents. These findings have important implications for the development of early interventions aimed at enhancing the well-being of adolescents. Considering the modifiable nature of unhealthy behaviors, it is crucial for adolescents to recognize the potential benefits of adopting healthy lifestyles. Therefore, we recommend implementing school and family-based interventions that promote healthy lifestyles as a preventive measure against self-injurious behavior and suicidality in adolescents.

### Supplementary Information

**Additional file 1: Table S1.** Prevalence of unhealthy behaviors among Chinese adolescents. **Table S2.** The weighted associations of different unhealthy behaviors with NSSI, suicidal ideation and suicidal attempt among Chinese adolescents.**Table S3.** The weighted associations of lifestyle risk score with NSSI, suicidal ideation and suicidal attempt among boys and girls.**Table S4.** The weighted associations of different unhealthy behaviors with NSSI, suicidal ideation and suicidal attempt among boys and girls.**Table S5.**. The Weighted associations of lifestyle risk score with NSSI, suicidal ideation and suicidal attempt among Chinese adolescents: using the complete dataset (n = 64176).**Figure S1.** The geographical distribution of the selected provinces.

## Data Availability

No datasets were generated or analysed during the current study.

## Abbreviations

NSSI: Non-suicidal self-injury
SI: Suicidal ideation
SA: Suicidal attempt
SCAHS: School-based Chinese Adolescents Health Survey
BMI: Body mass index
HSS: Household socioeconomic status
SD: Standard deviation
OR: Odds ratio
CI: Confidence interval
