# Supplementary material for: Prevalence of unhealthy behaviors and their associations with non-suicidal self-injury, suicidal ideation and suicide attempt among Chinese adolescents
Source: Child Adolesc Psychiatry Ment Health. 2024 May 29;18:61. doi: 10.1186/s13034-024-00742-y (PMC11137955; doi:10.1186/s13034-024-00742-y)
Supplement: Supplementary file 1 — Additional file 1: Table S1. Prevalence of unhealthy behaviors among Chinese adolescents. Table S2. The weighted associations of different unhealthy behaviors with NSSI, suicidal ideation and suicidal attempt among Chinese adolescents.Table S3. The weighted associations of lifestyle risk score with NSSI, suicidal ideation and suicidal attempt among boys and girls.Table S4. The weighted associations of different unhealthy behaviors with NSSI, suicidal ideation and suicidal attempt among boys and girls.Table S5.. The Weighted associations of lifestyle risk score with NSSI, suicidal ideation and suicidal attempt among Chinese adolescents: using the complete dataset (n = 64176).Figure S1. The geographical distribution of the selected provinces. [file 13034_2024_742_MOESM1_ESM.docx]

**Supplementary Information**

**Prevalence of** **unhealthy behaviors and their associations with non-suicidal self-injury, suicidal ideation and suicide attempt among Chinese adolescents**

**Table S1**. Prevalence of unhealthy behaviors among Chinese adolescents

**Table S2.** The weighted associations of different unhealthy behaviors with NSSI, suicidal ideation and suicidal attempt among Chinese adolescents

**Table S3.** The weighted associations of lifestyle risk score with NSSI, suicidal ideation and suicidal attempt among boys and girls

**Table S4.** The weighted associations of different unhealthy behaviors with NSSI, suicidal ideation and suicidal attempt among boys and girls

**Table S5.** The Weighted associations of lifestyle risk score with NSSI, suicidal ideation and suicidal attempt among Chinese adolescents: using the complete dataset (n = 64176).

**Figure S1.** The geographical distribution of the selected provinces.

| **Table S1. Prevalence of unhealthy behaviors among Chinese adolescents** | | | | |
| --- | --- | --- | --- | --- |
| Variable | Overall | Boys | Girls | *P* |
| Total | 74152 (100) | 36895 (100) | 37257 (100) |  |
| Insufficient physical activity |  |  |  |  |
| No | 16705 (23.7) | 10044 (27.8) | 6661 (19.1) | <0.001 |
| Yes | 57447 (76.3) | 26851 (72.2) | 30596 (80.9) |  |
| Current smoking |  |  |  |  |
| No | 70040 (95.3) | 33712 (92.9) | 36328 (98.1) | <0.001 |
| Yes | 4112 (4.7) | 3183 (7.1) | 929 (1.9) |  |
| Current drinking |  |  |  |  |
| No | 64368 (87.6) | 30508 (83.9) | 33860 (91.8) | <0.001 |
| Yes | 9784 (12.4) | 6387 (16.1) | 3397 (8.2) |  |
| Excessive screen time |  |  |  |  |
| No | 39871 (55.1) | 18773 (52.2) | 21098 (58.3) | <0.001 |
| Yes | 34281 (44.9) | 18122 (47.8) | 16159 (41.7) |  |
| Long homework time |  |  |  |  |
| No | 57111 (76.2) | 28834 (77.8) | 28277 (74.4) | <0.001 |
| Yes | 17041 (23.8) | 8061 (22.2) | 8980 (25.6) |  |
| Insufficient sleep |  |  |  |  |
| No | 26201 (36.8) | 14138 (40.0) | 12063 (33.2) | <0.001 |
| Yes | 47951 (63.2) | 22757 (60.0) | 25194 (66.8) |  |
| Unhealthy BMI |  |  |  |  |
| No | 54913 (74.8) | 26184 (71.8) | 28729 (78.1) | <0.001 |
| Yes | 19239 (25.2) | 10711 (28.2) | 8528 (21.9) |  |
| Lifestyle risk score |  |  |  |  |
| 0 | 2739 (4.1) | 1538 (4.6) | 1201 (3.5) | <0.001 |
| 1 | 11593 (16.7) | 5873 (17.0) | 5720 (16.4) |  |
| 2 | 22086 (30.0) | 10269 (28.4) | 11817 (31.8) |  |
| 3 | 21861 (29.0) | 10434 (27.8) | 11427 (30.3) |  |
| 4 | 11600 (15.0) | 6092 (15.6) | 5508 (14.3) |  |
| 5-7 | 4273 (5.2) | 2689 (6.5) | 1584 (3.8) |  |
| Notes: All numbers were unweighted, whereas all percentages were adjusted for sampling weights | | | | |

| **Table S2. The weighted associations of different unhealthy behaviors with NSSI, suicidal ideation and suicidal attempt among Chinese adolescents** | | | | | | |
| --- | --- | --- | --- | --- | --- | --- |
| **Outcome** | **Unhealthy behavior** | **Weighted prevalence of outcomes, %** | **Model 1** | | **Model 2** | |
|  |  |  | **OR (95% CI)** | ***P*** | **OR (95% CI)** | ***P*** |
| **NSSI** |  |  |  |  |  |  |
|  | Insufficient physical activity | 15.0 (14.6-15.3) | 1.08 (1.02, 1.15) | 0.008 | 1.06 (1.00, 1.13) | 0.047 |
|  | Current smoking | 22.5 (21.0-24.0) | 2.30 (2.10, 2.52) | <0.001 | 2.14 (1.94, 2.35) | <0.001 |
|  | Current drinking | 24.6 (23.6-25.6) | 2.62 (2.46, 2.79) | <0.001 | 2.55 (2.39, 2.72) | <0.001 |
|  | Excessive screen time | 16.9 (16.5-17.4) | 1.51 (1.44, 1.59) | <0.001 | 1.52 (1.45, 1.60) | <0.001 |
|  | Long homework time | 18.2 (17.5-18.8) | 1.44 (1.36, 1.52) | <0.001 | 1.46 (1.38, 1.54) | <0.001 |
|  | Insufficient sleep | 17.4 (17.1-17.8) | 2.09 (1.97, 2.21) | <0.001 | 2.04 (1.92, 2.16) | <0.001 |
|  | Unhealthy BMI | 14.9 (14.3-15.5) | 1.09 (1.03, 1.15) | 0.003 | 1.09 (1.04, 1.16) | 0.001 |
| **Suicide ideation** |  |  |  |  |  |  |
|  | Insufficient physical activity | 18.2 (17.8-18.5) | 1.13 (1.07, 1.20) | <0.001 | 1.11 (1.05, 1.18) | <0.001 |
|  | Current smoking | 28.7 (27.1-30.4) | 2.76 (2.54, 3.00) | <0.001 | 2.68 (2.46, 2.93) | <0.001 |
|  | Current drinking | 29.4 (28.3-30.5) | 2.82 (2.65, 2.99) | <0.001 | 2.76 (2.60, 2.93) | <0.001 |
|  | Excessive screen time | 21.4 (20.9-21.9) | 1.79 (1.71, 1.87) | <0.001 | 1.78 (1.70, 1.86) | <0.001 |
|  | Long homework time | 20.5 (19.8-21.3) | 1.30 (1.24, 1.37) | <0.001 | 1.29 (1.23, 1.36) | <0.001 |
|  | Insufficient sleep | 20.8 (20.4-21.3) | 2.06 (1.95, 2.17) | <0.001 | 1.99 (1.89, 2.10) | <0.001 |
|  | Unhealthy BMI | 18.4 (17.7-19.0) | 1.14 (1.08, 1.20) | <0.001 | 1.16 (1.10, 1.22) | <0.001 |
| **Suicide attempt** |  |  |  |  |  |  |
|  | Insufficient physical activity | 5.3 (5.1-5.5) | 1.06 (0.96, 1.16) | 0.231 | 1.03 (0.94, 1.13) | 0.550 |
|  | Current smoking | 13.0 (11.8-14.2) | 4.73 (4.19, 5.34) | <0.001 | 4.38 (3.86, 4.98) | <0.001 |
|  | Current drinking | 11.7 (10.9-12.4) | 4.05 (3.70, 4.43) | <0.001 | 3.92 (3.58, 4.30) | <0.001 |
|  | Excessive screen time | 6.6 (6.3-7.0) | 1.92 (1.78, 2.07) | <0.001 | 1.91 (1.77, 2.07) | <0.001 |
|  | Long homework time | 5.6 (5.2-6.0) | 1.15 (1.05, 1.26) | 0.002 | 1.19 (1.09, 1.30) | <0.001 |
|  | Insufficient sleep | 6.2 (5.9-6.4) | 1.98 (1.81, 2.17) | <0.001 | 1.94 (1.77, 2.13) | <0.001 |
|  | Unhealthy BMI | 5.6 (5.3-6.0) | 1.18 (1.08, 1.28) | <0.001 | 1.17 (1.07, 1.28) | <0.001 |
| Model 1 adjusted for age, sex  Model 2 adjusted for age, sex, province, residence, having brothers/sisters, living arrangement, HSS, and parents' education level  Abbreviations: NSSI, Non-suicidal Self-Injury | | | | | | |

| **Table S3. The weighted associations of lifestyle risk score with NSSI, suicidal ideation and suicidal attempt among boys and girls** | | | | | | |
| --- | --- | --- | --- | --- | --- | --- |
| **Outcome** | **Lifestyle risk score** | **Model 2, OR (95% CI)** | | | **ROR** ^b^ | ***P* value ^c^** |
|  |  | **Overall** | **Boys** | **Girls** |  |  |
| **NSSI** |  |  |  |  |  |  |
|  | 0 | 1.00 (reference) | 1.00 (reference) | 1.00 (reference) | NA | NA |
|  | 1 | 1.47 (1.21, 1.78) | 1.45 (1.07, 1.95) | 1.49 (1.16, 1.90) | 1.03 | 0.446 |
|  | 2 | 2.21 (1.84, 2.65) | 2.17 (1.63, 2.90) | 2.25 (1.78, 2.84) | 1.04 | 0.429 |
|  | 3 | 3.37 (2.81, 4.05) | 3.22 (2.42, 4.29) | 3.50 (2.77, 4.43) | 1.09 | 0.329 |
|  | 4 | 4.57 (3.80, 5.51) | 4.27 (3.19, 5.72) | 4.79 (3.77, 6.10) | 1.12 | 0.276 |
|  | 5-7 | 6.38 (5.24, 7.77) | 5.15 (3.80, 6.98) | 7.95 (6.12, 10.32) | 1.54 | 0.017 |
| **Suicide ideation** |  |  |  |  |  |  |
|  | 0 | 1.00 (reference) | 1.00 (reference) | 1.00 (reference) | NA | NA |
|  | 1 | 1.43 (1.19, 1.71) | 1.27 (0.95, 1.69) ^a^ | 1.55 (1.22, 1.96) | 1.22 | 0.146 |
|  | 2 | 2.33 (1.95, 2.77) | 2.33 (1.77, 3.05) | 2.35 (1.87, 2.95) | 1.01 | 0.479 |
|  | 3 | 3.49 (2.93, 4.15) | 3.18 (2.43, 4.17) | 3.73 (2.98, 4.68) | 1.17 | 0.186 |
|  | 4 | 5.14 (4.30, 6.15) | 4.72 (3.58, 6.21) | 5.48 (4.34, 6.91) | 1.16 | 0.208 |
|  | 5-7 | 7.67 (6.35, 9.25) | 6.18 (4.64, 8.22) | 9.75 (7.57, 12.55) | 1.58 | 0.010 |
| **Suicide attempt** |  |  |  |  |  |  |
|  | 0 | 1.00 (reference) | 1.00 (reference) | 1.00 (reference) | NA | NA |
|  | 1 | 1.34 (0.97, 1.84) | 1.08 (0.61, 1.90) ^a^ | 1.46 (0.99, 2.15) ^a^ | 1.35 | 0.192 |
|  | 2 | 2.32 (1.71, 3.15) | 2.44 (1.43, 4.17) | 2.27 (1.57, 3.28) | 0.93 | 0.413 |
|  | 3 | 3.57 (2.63, 4.84) | 3.18 (1.86, 5.43) | 3.78 (2.62, 5.46) | 1.19 | 0.301 |
|  | 4 | 5.46 (4.00, 7.44) | 5.86 (3.41, 10.05) | 5.19 (3.56, 7.54) | 0.89 | 0.358 |
|  | 5-7 | 9.57 (6.95, 13.17) | 7.31 (4.20, 12.72) | 11.67 (7.90, 17.24) | 1.60 | 0.088 |
| Notes: Model 2 adjusted for age, sex, province, residence, having brothers/sisters, living arrangement, HSS, and parents' education level (models for boys and girls were not adjusted for sex)  ^a^ *P* > 0.05  ^b^ Calculated by OR of girls / OR of boys  ^c^ A one-tailed P-value  Abbreviations: NSSI, Non-suicidal Self-Injury; NA, not applicable | | | | | | |

| **Table S4. The weighted associations of different unhealthy behaviors with NSSI, suicidal ideation and suicidal attempt among boys and girls** | | | | | | |
| --- | --- | --- | --- | --- | --- | --- |
| **Outcome** | **Unhealthy behavior** | **Model 2, OR (95% CI)** | | | **ROR** ^b^ | ***P* value ^c^** |
|  |  | **Overall** | **Boys** | **Girls** |  |  |
| **NSSI** |  |  |  |  |  |  |
|  | Insufficient physical activity | 1.06 (1.00, 1.13) | 1.04 (0.96, 1.14) ^a^ | 1.08 (1.00, 1.17) ^a^ | NA | NA |
|  | Current smoking | 2.14 (1.94, 2.35) | 1.50 (1.32, 1.71) | 4.40 (3.75, 5.17) | 2.93 | <0.001 |
|  | Current drinking | 2.55 (2.39, 2.72) | 1.97 (1.80, 2.16) | 3.37 (3.08, 3.69) | 1.71 | <0.001 |
|  | Excessive screen time | 1.52 (1.45, 1.60) | 1.48 (1.37, 1.60) | 1.56 (1.46, 1.66) | 1.05 | 0.157 |
|  | Long homework time | 1.46 (1.38, 1.54) | 1.55 (1.43, 1.69) | 1.40 (1.30, 1.50) | 0.90 | 0.030 |
|  | Insufficient sleep | 2.04 (1.92, 2.16) | 2.04 (1.87, 2.23) | 2.03 (1.89, 2.19) | 1.00 | 0.469 |
|  | Unhealthy BMI | 1.09 (1.04, 1.16) | 1.07 (0.98, 1.16) ^a^ | 1.11 (1.03, 1.20) | 1.04 | 0.241 |
| **Suicide ideation** |  |  |  |  |  |  |
|  | Insufficient physical activity | 1.11 (1.05, 1.18) | 1.15 (1.06, 1.25) | 1.08 (1.00, 1.17) | 0.94 | 0.147 |
|  | Current smoking | 2.68 (2.46, 2.93) | 2.14 (1.90, 2.40) | 5.15 (4.38, 6.05) | 2.41 | <0.001 |
|  | Current drinking | 2.76 (2.60, 2.93) | 2.10 (1.92, 2.29) | 3.89 (3.56, 4.24) | 1.85 | <0.001 |
|  | Excessive screen time | 1.78 (1.70, 1.86) | 1.67 (1.55, 1.80) | 1.86 (1.75, 1.97) | 1.11 | 0.013 |
|  | Long homework time | 1.29 (1.23, 1.36) | 1.29 (1.19, 1.41) | 1.29 (1.21, 1.38) | 1.00 | 0.491 |
|  | Insufficient sleep | 1.99 (1.89, 2.10) | 2.09 (1.92, 2.28) | 1.93 (1.80, 2.07) | 0.92 | 0.072 |
|  | Unhealthy BMI | 1.16 (1.10, 1.22) | 1.14 (1.05, 1.23) | 1.17 (1.10, 1.26) | 1.03 | 0.280 |
| **Suicide attempt** |  |  |  |  |  |  |
|  | Insufficient physical activity | 1.03 (0.94, 1.13) ^a^ | 1.00 (0.86, 1.17) ^a^ | 1.05 (0.93, 1.18) ^a^ | NA | NA |
|  | Current smoking | 4.38 (3.86, 4.98) | 3.46 (2.84, 4.20) | 6.44 (5.41, 7.68) | 1.86 | <0.001 |
|  | Current drinking | 3.92 (3.58, 4.30) | 3.13 (2.68, 3.65) | 4.62 (4.12, 5.17) | 1.48 | <0.001 |
|  | Excessive screen time | 1.91 (1.77, 2.07) | 1.94 (1.68, 2.24) | 1.91 (1.73, 2.09) | 0.98 | 0.410 |
|  | Long homework time | 1.19 (1.09, 1.30) | 1.22 (1.04, 1.44) | 1.17 (1.05, 1.30) | 0.96 | 0.327 |
|  | Insufficient sleep | 1.94 (1.77, 2.13) | 2.04 (1.73, 2.42) | 1.90 (1.70, 2.12) | 0.93 | 0.234 |
|  | Unhealthy BMI | 1.17 (1.07, 1.28) | 1.10 (0.94, 1.28) ^a^ | 1.20 (1.08, 1.33) | 1.09 | 0.171 |
| Notes: Model 2 adjusted for age, sex, province, residence, having brothers/sisters, living arrangement, HSS, and parents' education level (models for boys and girls were not adjusted for sex)  ^a^ *P* > 0.05  ^b^ Calculated by OR of girls / OR of boys  ^c^ A one-tailed P-value  Abbreviations: NSSI, Non-suicidal Self-Injury; NA, not applicable | | | | | | |

| **Table S5. The weighted associations of lifestyle risk score with NSSI, suicidal ideation and suicidal attempt among Chinese adolescents: using the complete dataset (n = 64176).** | | | | | | |
| --- | --- | --- | --- | --- | --- | --- |
| **Outcome** | **Lifestyle risk score** | **Model 1** | | **Model 2** | | ***P* for trend*** |
|  |  | **OR (95% CI)** | ***P*** | **OR (95% CI)** | ***P*** |  |
| **NSSI** |  |  |  |  |  | <0.001 |
|  | 0 | 1.00 (reference) |  | 1.00 (reference) |  |  |
|  | 1 | 1.50 (1.24, 1.82) | <0.001 | 1.45 (1.18, 1.78) | <0.001 |  |
|  | 2 | 2.30 (1.92, 2.76) | <0.001 | 2.22 (1.82, 2.70) | <0.001 |  |
|  | 3 | 3.52 (2.93, 4.22) | <0.001 | 3.34 (2.74, 4.06) | <0.001 |  |
|  | 4 | 4.81 (3.99, 5.79) | <0.001 | 4.48 (3.67, 5.47) | <0.001 |  |
|  | 5-7 | 6.88 (5.65, 8.38) | <0.001 | 6.30 (5.10, 7.79) | <0.001 |  |
| **Suicide ideation** |  |  |  |  |  | <0.001 |
|  | 0 | 1.00 (reference) |  | 1.00 (reference) |  |  |
|  | 1 | 1.45 (1.21, 1.74) | <0.001 | 1.40 (1.15, 1.70) | 0.001 |  |
|  | 2 | 2.42 (2.03, 2.88) | <0.001 | 2.30 (1.91, 2.77) | <0.001 |  |
|  | 3 | 3.67 (3.09, 4.37) | <0.001 | 3.46 (2.87, 4.17) | <0.001 |  |
|  | 4 | 5.45 (4.56, 6.50) | <0.001 | 5.17 (4.28, 6.26) | <0.001 |  |
|  | 5-7 | 8.24 (6.83, 9.95) | <0.001 | 7.45 (6.09, 9.10) | <0.001 |  |
| **Suicide attempt** |  |  |  |  |  | <0.001 |
|  | 0 | 1.00 (reference) |  | 1.00 (reference) |  |  |
|  | 1 | 1.38 (1.00, 1.90) | 0.051 | 1.35 (0.95, 1.92) | 0.097 |  |
|  | 2 | 2.43 (1.79, 3.29) | <0.001 | 2.40 (1.72, 3.35) | <0.001 |  |
|  | 3 | 3.74 (2.76, 5.08) | <0.001 | 3.70 (2.65, 5.16) | <0.001 |  |
|  | 4 | 5.81 (4.26, 7.91) | <0.001 | 5.56 (3.96, 7.80) | <0.001 |  |
|  | 5-7 | 10.50 (7.63, 14.45) | <0.001 | 9.55 (6.74, 13.55) | <0.001 |  |
| Model 1 adjusted for age, sex  Model 2 adjusted for age, sex, province, residence, having brothers/sisters, living arrangement, HSS, and parents' education level  **P* for trend was estimated based on Model 2  Abbreviations: NSSI, Non-suicidal Self-Injury | | | | | | |


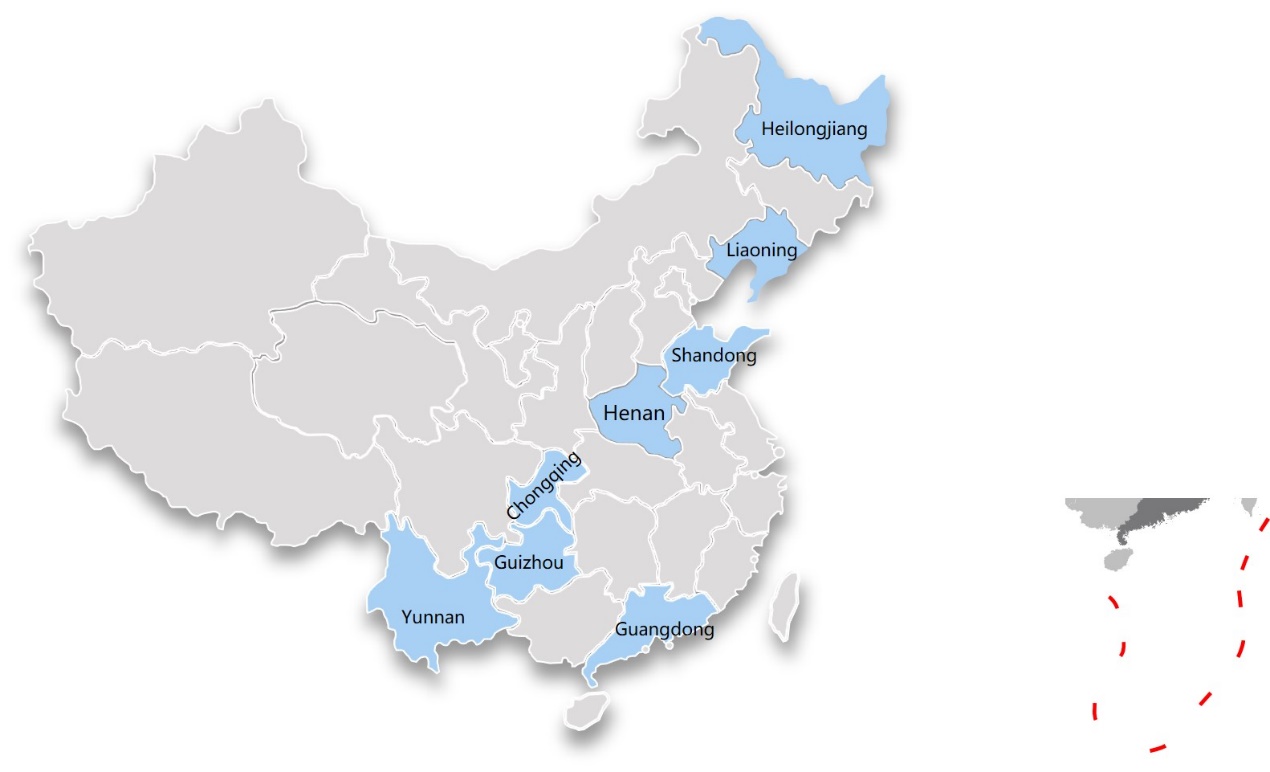


**Figure S1. The geographical distribution of the selected provinces.**
